# Promoting wellbeing and health through active participation in music and dance: a systematic review

**DOI:** 10.1080/17482631.2020.1732526

**Published:** 2020-04-08

**Authors:** Alexa Sheppard, Mary C. Broughton

**Affiliations:** a School of Public Health, The University of Queensland, Brisbane, Australia; b School of Music, The University of Queensland, Brisbane, Australia

**Keywords:** Music, dance, active participation, wellbeing, population health, social determinants of heath

## Abstract

**Purpose**: This review aims to reveal how music and dance participation relates to key social determinants of health, and acts as population wellbeing and health promotion and preventive tools for people without pre-existing health issues in diverse social groups.

**Methods**: A qualitative systematic literature review addresses the questions: 1) How might music and dance cultural practices relate to social determinants of health?; 2) What gaps exist in the current body of research examining how healthy individuals/populations maintain and promote good health and wellbeing through music and dance participation?; 3) What are the implications of the current body of literature for healthcare, policy and research?

**Results**: Music and dance relate to key social determinants of health, from social and cultural, and physical and mental health perspectives. A number of gaps in the literature exist, including underrepresentation of middle aged participants, men, and Indigenous, LGBTQIAP+, and migrant populations. There is a lack of consistency in theoretical and methodological approaches, and a language for effective communication across arts and health audiences.

**Conclusions**: A significant opportunity exists for cross-disciplinary collaboration to advance healthcare and arts policy, generate cost-effective approaches to preventive healthcare practice, and enhance the wellbeing and health of large and diverse populations.

Wellbeing is an important topic for individuals, societies and public policy around the world. It is increasingly being viewed as an important component for policy makers for its impact on economic, health, social and cultural facets of life (Adler & Seligman, 2016). In the modern world, perceptions of what it means to be healthy indicate an imperative to explore alternative ways of addressing quality of life and wellbeing for all people, at all stages of life and levels of health. Performing arts are a social praxis offering potential wellbeing and health benefits to participants, and are increasingly being viewed as an alternative or complement to traditional biomedical health practices and processes (Batt-Rawden, 2010; Edwards, 2011; Kreutz, 2008; MacDonald, Kreutz, & Mitchell, 2012; Stewart & Irons, 2018). The aim of this systematic review is to synthesize research on performing arts and wellbeing and health. We focus particularly on active participation and healthy populations, taking a social determinants of health perspective to examine how in today’s world humans are maintaining their wellbeing and health through performing arts that involve music.

Throughout history, music and dance have been used consistently as tools for healing and health similar to the way that pharmacology and therapies are used today (Stewart & Irons, 2018). Examples of this include the use of music as therapy or treatment in ancient Islamic civilizations (Sufie & Sidik, 2017), shamanic dance healing in Siberia and other European and American countries, and the ancient Greek scholar Pythagoras using music to soothe distressed organs (Stewart & Irons, 2018). Equally, the performing arts have historically been cited as making people “feel good” and improving their “quality of life”. Historically, the performing arts have been vitally important not only to individuals in ill-health, but also an important contributor to the wellbeing and health maintenance of individuals without pre-existing health, behavioural, or social issues. While the strength of connection between performing arts and health seems to have diminished in modern times, particularly in Western societies, there is growing interest in applying a modern-day lens to understand the contribution of performing arts participation to wellbeing and health.

Arts health is a domain concerned with promoting positive wellbeing and health outcomes for participants through engagement with various art forms, including music and dance (MacDonald et al., 2012). It resides in the theoretical space where art and science meet. There have been many scientific, phenomenological, philosophical, and salutogenic studies conducted in recent times suggesting that arts and health might have more in common than is generally accepted by health professionals and policymakers (Bungay & Vella-Burrows, 2013; Cacioppo & Hawkley, 2003; Crawford, Brown, Baker, Tischler, & Abrams, 2015; Dawson, 2013; Edwards, 2011; Evans, 2007; Wreford, 2010). For example, healthcare and performing arts, particularly music and dance practices, share three defining features: that they are relational, aesthetic, and temporal (Crawford et al., 2015). In healthcare and artistic domains, the environment is crucially connected to and shapes health or artistic outcomes (*relational*). The individual or population perception of key concepts, such as what is “good health” or “good music”, reflects an *aesthetic* perception. The *temporal* element acknowledges that both health and artistic states unfold in time within the context of people’s lived experience. With such common ground, both being practices created by humans for humans, it is plausible that human experiences of healthcare and performing arts practices might also share some positive wellbeing and health outcomes for participants. Despite this, the arts have largely gone unrecognized or been dismissed by many contemporary health science professionals and policymakers (Owen, 1999). However, a recent, emerging body of research is examining the contribution of performing arts to positive health outcomes for clinical populations.

Recent research on arts and health centres on how performing arts can best serve people living with debilitating and chronic illness such as dementia, Parkinson’s disease, and mental illness (Adam, Ramli, & Shahar, 2016; Coaten & Newman-Bluestein, 2013; Guzman, Freeston, Rochester, Hughes, & James, 2016; Jones, 2016; Karkou & Meekums, 2017; Lannen, Worrall, Alston, Marley, & Allan, 2012; Lapum & Bar, 2016; Särkämö et al., 2016; Sorrell, 2018; Sung & Chang, 2005; Sung, Chang, & Lee, 2010; Verghese et al., 2006). For example, multiple studies have shown that dance therapy can significantly improve gait, movement, balance, and wellbeing in patients with Parkinson’s disease (Butt, 2017; Hackney & Earhart, 2010; Hackney & McKee, 2014; Hashimoto, Takabatake, Miyaguchi, Nakanishi, & Naitou, 2015; Karkou & Meekums, 2017; Lewis, Annett, Davenport, Hall, & Lovatt, 2016; McNeely, Duncan, & Earhart, 2015; Rios Romenets, Anang, Fereshtehnejad, Pelletier, & Postuma, 2015; Rocha, Slade, McClelland, & Morris, 2017; Shanahan et al., 2017; Ventura et al., 2016). Music therapy is also a well-established approach to treating a range of issues, such as speech pathologies, poor mental health, and social and behavioural difficulties in children and adults (Edwards, 2011; MacDonald et al., 2012). A defining factor of these approaches, however, is their focus on disease/disorder treatment rather than health maintenance in non-clinical populations, which is the focus of the present review.

Performing arts are defined as any creative arts practice that is performed in front of audience, including music, dance, and drama (Macmillan, n.d.; Oxford, n.d.). Here, we consider performing arts as socio-cultural practices in which participants engage actively, rather than experience passively as an observer. We acknowledge that this definition is Western-centric. In some non-Western cultural practices, concepts of music and dance are inseparable (e.g., *ngoma* in Africa), tied to other culturally important concepts (e.g., kinship, ceremony, and country for Indigenous Australians, see Spillman, 2018), or not designed for performing in front of an audience, but are none-the-less social and community-based. This review is therefore inclusive of activities that involve active participation in a cultural practice that includes music and/or dance. Given the active, social and cultural basis for performing arts, it is plausible that these practices, particularly music and dance, might crucially relate to social determinants of health (Stewart & Irons, 2018). Social determinants of health (Wilkinson & Marmot, 2003) are a facet of a fundamental concept in public health that a person’s good or ill health is determined by social, cultural, political, and environmental factors, some of which are within their control, and some that are not. According to the World Health Organization (WHO), wellbeing is a crucial aspect of health. Marmot (2011, p. 42) describes wellbeing as “a multidimensional construct, which includes satisfaction with life, a sense of autonomy, control and self-realisation, and the absence of depression and loneliness”. Therefore, active engagement in socio-cultural performing arts practices, such as music and dance, have the potential to enhance, improve, and maintain wellbeing, with likely impact on the quality of health experienced by individuals and social groups.

A contemporary understanding of health and what it means to be healthy needs to incorporate more than medical or biological perspectives. We need to develop our understanding of the ways in which populations maintain good health and prevent ill health, especially when it comes to equipping us to deal with the complex and prevalent issues involved in psychological and physical health. Future global health issues, such as the rapidly growing ageing population and the growing number of people (particularly in Western-centred cultures) suffering from health problems, such as depression and obesity, might both be addressed using performing arts interventions. Reviewing the impacts that active engagement in cultural activities such as music and dance can have on populations and communities has the potential to identify, prevent and improve health and enhance quality of life in populations in ways that are both cost effective and enjoyable.

## Research aim and questions

The aim of this paper is to review contemporary literature addressing the effectiveness of performing arts, specifically music and dance participation, to maintaining and enhancing good health and wellbeing within a population. Specifically, we address the following key questions:

- How might music and dance cultural practices relate to social determinants of health?
- What gaps exist in the current body of research examining how healthy individuals/populations maintain and promote good health and wellbeing through music and dance participation?
- What are the implications of the current body of literature for healthcare, policy and research?

## Methods

### Search strategy

A literature search was undertaken in September 2018 to locate research examining wellbeing and health through music and dance. To find studies to review the PubMed and EMBASE databases were utilized. The MeSH terms *music* (also replaced with *singing*), or *dance* were combined with terms such as *creativity, health, wellbeing, quality of life, social participation/isolation/support, prevention*, and “*health promotion*”. Because of the multidisciplinary nature of this subject, prominent sources in other disciplinary areas, such as the Journal of Music Therapy and Journal of Arts and Health, were searched specifically for resources. Additionally, review papers and systematic reviews related to the broad topic area were used as guides to source other studies that fit the inclusion criteria.

**Figure 1.**
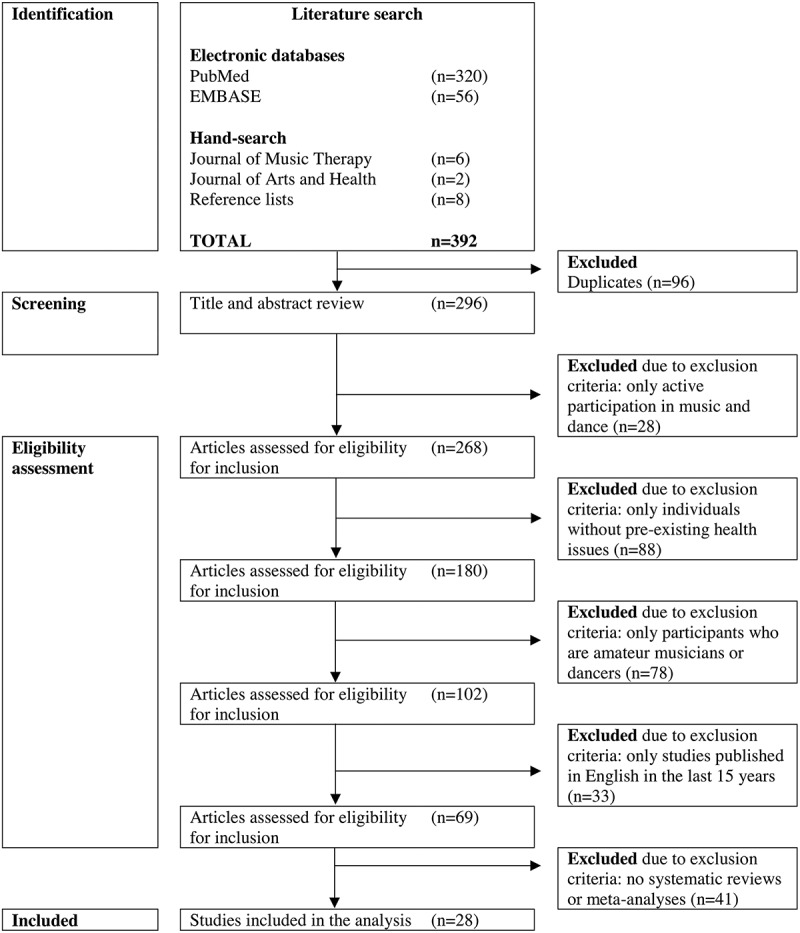
Flowchart of the literature identification and selection process

The studies relevant to this review were selected by identifying key words and concepts within individual titles and abstracts. Studies that met the inclusion criteria (detailed below) were categorized into two different groups based on their relevance to music or dance. Studies that did not meet the inclusion criteria or met the exclusion criteria were discarded. Figure 1 presents a flowchart of the process of identifying and selecting literature. Data was extracted by close reading of the shortlisted studies and then summarized in tables (see Tables I-IV for detail). For inclusion in this review, each selected study had to have been subject to peer review processes prior to publication, and had to present a clear, consistent methodology, which were both taken as indicators of research quality. Due to the nature of music and dance, it was expected that much of the data gathered would be either qualitative or use a mixed methods approach. Because of this, the review is presented here as a qualitative summary of key themes, concepts and results identified in the research.

**Table I.** Summary of Participant Age Groups, Musical Activity, Research Design, Methodological Approaches, and Outcome Examined

	Musical Activity				Research Design						Methodological Approach			Outcome Examined		
Age Group	Singing	Instr. Music	Various*	Previous Experience	Pre-Post Test	Long.	RCT	Case Study	Ethno.	Comp.	Qual.	Quant.	Mixed	Wellbeing	Cog. Health	Total Studies for Age Group
Adults >60 years	1	2	1	1	2	1	1			1		4	1	3	2	5
Adults >43 years		1			1								1	1		1
“Older” adults	1							1			1			1		1
Adults 18–43 years																0
Children <18 years			1		1						1			1		1
Unspecified	1								1		1			1		1
Total	3	3	2	1	4	1	1	1	1	1	3	4	2	7	2	

Instr. = Instrumental (music), Ethno. = Ethnomusicological, Comp. = Comparative, Long. = Longitudinal, Qual. = Qualitative, Quant. = Quantitative, Cog. = Cognitive (health).

*Various includes a range of activities, for example: Listening, singing, moving, song-writing, or music appreciation.

**Table II.** Detailed Summary of the Music Research Results

Citation	Wellbeing/Cognitive Health	Participant Demographics	Activity	Study Design/ Measurement/ Measurement Tool/ Type of Data	Results/Key Concepts
Cohen et al. (2006)	Wellbeing	N = 166 (90 in intervention group) Age >65 years 78% female Washington DC, USA	Choir	Part of a larger longitudinal study Overall health Social health Depression/Loneliness Questionnaires/self-report Quantitative	Intervention group reported increase in overall health and social health
Creech et al. (2013)	Wellbeing	N = 500 (81% female) Age 43–93 (mode 65 years) UK	Instrumental music	Pre-test/post-test Autonomy Competence Relatedness Self-Realization Questionnaires Interviews Mixed methods	Positive changes
Joseph and Southcott (2014)	Wellbeing	Older Australians (age unspecified) 2 interviewees (1 male, 1 female) Melbourne, Australia	Choir	Case study Qualitative	Positive changes
Kim et al. (2006)	Wellbeing	N = 35 Age 11–12 years Girls Korea	Music participation	Post-test Positive social relationships Perceived self-control Direct observations Self-report Qualitative	Positive changes
McConnell (2016)	Wellbeing	The Gambia Age unspecified	Singing	Ethnomusicological study Qualitative	Positive changes
Solé et al. (2010)	Wellbeing	N = 83 (69 women) Mean age ~72.6 years Barcelona, Spain	Three music-based activities including a choir, music appreciation class, and a preventive music therapy programme	Pre-test/post-test Quality of Life Researcher-designed questionnaires Mixed methods	No significant changes
Yap et al. (2017)	Wellbeing	N = 31 (94% female) Age >65 years Singapore	Rhythm-centred music making programme	Randomized control trial with cross-over Depression Sleep quality Social isolation Questionnaires Quantitative	No significant changes
Bugos et al. (2007)	Cognition	N = 39 (16 intervention group) Age 60–85 years Gainesville, USA	Piano	Pre-test/post-test Working memory Executive function Cognitive performance tests Quantitative	Positive changes
Hanna-Pladdy and MacKay (2011)	Music (cognition)	N = 70 50–60% female for each group of participants) Age 60–83 years USA	Previous musical experience (non-professional, under 10 years)	Comparative study Sensorimotor and cognitive abilities as transferrable skills (to non-music activities) Cognitive performance tests Quantitative	Positive result—people with more musical training performed better than people without/with less music training

**Table III.** Summary of Participant Age Groups, Dance Activity, Research Design, Methodological Approaches, and Outcome Examined

	Dance Activity					Research Design					Methodological Approach			Outcome Examined				
Age Group	Con- temp.	Cul- tural *	Unspeci- fied	Partnered **	Var- ious ***	Pre-Post Test	RCT	Quasi-Expt.	Ethno.	Comp.	Qual.	Quant.	Mixed	Wellbeing	Cog. Hlth	Phys. Hlth	Cog. and Phys. Hlth	Total Studies for Age Group
Adults >60 years	1	3	3	3	1	3	3		1	4	1	9	1	2	1	6	2	11
Adults >50 years		1							1		1			1				1
Adults 18–82 years	1			1			1	1				2		1		1		2
Adults 18–60 years	1	1		1		2			1		1	1	1	2		1		3
Children <18 years	1			1		1	1				1	1		1		1		2
Total	4	5	3	6	1	6	5	1	3	4	4	13	2	7	1	9	2	

Contemp. = Contemporary (dance), Expt. = Experimental, Ethno. = Ethnographic/Observational/Exploratory, Comp. = Comparative, Long. = Longitudinal, Qual. = Qualitative, Quant. = Quantitative, Cog. Hlth = Cogntive Health, Phys. hlth = Physical Health.

*Cultural dance includes Scottish country dance, belly dancing, Colombian Caribbean folk dancing, and line dancing

**Partnered dance includes salsa, tango, and ballroom dancing

***Various includes a combination of dance activities including folk dances or ballroom dance.

**Table IV.** Detailed Summary of the Dance Research Results

Citation	Wellbeing/Cognition/Physical Health	Participant Demographics	Activity	Study Design/ Measurement/ Measurement Tool/ Type of Data	Results/Key Concepts
Coubard et al. (2011)	Cognition	N = 110 (104 females) Age 65–83 years France	Dance vs. fall prevention classes vs. tai chi	Comparative study Attention switching Cognitive flexibility Performance tests Quantitative	Positive improvement in dance group compared with other groups
Cruz-Ferreira et al. (2015)	Wellbeing	N = 57 women 65–80 years Portugal	Contemporary dance	Randomized control trial Physical fitness Life satisfaction Questionnaire Fitness test Quantitative	Positive improvement in intervention group
Dewhurst et al. (2014)	Physical health	N = 60 women Average age ~67.6 years Scotland	Scottish country dancing	Comparative study Functional ability Balance Performance tests Quantitative	Positive change in intervention group
Duberg et al. (2016)	Wellbeing	N = 112 girls Age 13–18 years Sweden	Contemporary dance	Randomized control study Emotional expression Social competence Self-report diary Interview Qualitative	Positive associations with dance
Filar-Mierzwa et al. (2017)	Physical health	N = 24 All women Age 61–74 years Poland	Dance	Pre-test/post-test Balance Posture Fall prevention Performance test Quantitative	Positive improvement in dance group
Granacher et al. (2012)	Physical health	N = 28 (17 female) Age 63–82 years Germany	Salsa dancing	Randomized control trial Static/dynamic postural control Leg extensor power Performance tests Questionnaire Quantitative	Positive improvement in dance group
Huang et al. (2012)	Physical health	N = 79 (across 2 primary schools) 8–11 years New York	Ballroom dancing	Pre-test/post-test Time involved in physical activity Heart rate Direct monitoring/observation Biometric data Quantitative	Positive improvement post-test
Jan-Christoph et al. (2010)	Cognition and physical health	N = 62 (49 female) Age 61–94 years Germany	Ballroom dance	Comparative study Everyday competence Cognitive performance Multiple-choice reaction time measurement Motor and tactile performance Performance tests Quantitative	Positive changes in intervention group
Kattenstroth et al. (2013)	Cognition and physical health	N = 35 (25 women) Age 60–94 years Germany	Dance class (specifically designed for aged participants)	Pre-test/post-test Posture Motor performance Cognitive performance Tactile performance Reaction time Performance tests Quantitative	Positive change in intervention group
Kreutz (2008)	Wellbeing	N = 110 (41.4% female) Age ~37.96 years Netherlands and Germany	Ballroom dancing	Exploratory Interview Participant observation Qualitative	Positive associations with dance
Merom et al. (2016)	Physical health	N = 530 (across 23 self-care retirement villages) 85% female Mean age 78 years old Sydney, Australia	Social dance class (folk or ballroom dancing)	Randomized control trial Falls Fall related mechanisms Self-report Performance tests Mixed methods	No improvement
Moe (2014)	Wellbeing	N = 16 women Age >50 years USA	Belly dancing	Participant observation Interviews Qualitative	Positive
Muro and Artero (2017)	Wellbeing	N = 201 women Age ~20.88 years Spain	Modern dance	Pre-test/post-test Life satisfaction Mindfulness Questionnaire Quantitative	Positive associations
Murrock and Gary (2008)	Physical health	N = 126 women Age 36–82 years USA	Contemporary dance	Quasi-experimental Functional capacity Performance test Quantitative	Positive improvement in intervention group
Nadasen (2008)	Wellbeing	N = 30 women Age 60–82 years South Africa	Line dancing	Participant observation Social activity Interview Qualitative	Positive improvement
Pacheco et al. (2016)	Physical health	N = 27 women Age >60 years Colombian Caribbean region	Colombian Caribbean folk dance	Pre-test/post-test Cardiorespiratory function Strength Balance Direct observation Quantitative	Positive improvement in intervention group
Pinniger et al. (2012)	Wellbeing	N = 66 (90.0% female) Age 18–80 years Australia	Tango vs. meditation vs. control	Randomized control trial Depression, anxiety and stress Self-esteem Life satisfaction Mindfulness Questionnaire Self-report Quantitative	Positive improvement in mindfulness but not self-esteem or life satisfaction
Rahal et al. (2015)	Physical health	N = 76 (56% female) Age >60 years Brazil	Ballroom dancing vs. tai chi	Comparative study Posture Balance Performance tests Quantitative	Positive improvements for both groups
Vahabi and Damba (2015)	Physical health	N = 30 Women Age 22–58 years South Asian migrants in Toronto, Canada	Bollywood dancing	Pre-test/post-test Physical activity Physical health Questionnaire Interviews Biometric data Mixed methods	Positive improvement post-test

### Inclusion and exclusion criteria

Studies that focussed on music and dance participation were included. Theatre participation was excluded because it is not as readily accessible to all population groups as are music and dance, which are fundamental performing arts practices people can engage in using only the human voice and body. Only studies that focussed on individuals actively participating in music and dance were included. Studies that focused on passive listening to music or watching a music/dance performance were not included. Additionally, aerobic dance practices for physical fitness were also excluded from this review as they were considered to be primarily representative of sport or exercise practices rather than creative performing arts.

Only studies that focussed on individuals without pre-existing health issues were included. Studies that looked at the benefits of music and dance to participants with neurological, physiological or psychological impairments were excluded. Studies focusing on participants with Parkinson’s disease, dementia, clinically diagnosed mental health disorders such as depression, anxiety, schizophrenia, personality disorders, and cognitive, behavioural, and intellectual disorders such as autism spectrum disorder, and chronic physical diseases such as cardiovascular diseases were excluded.

The inclusion criteria did not specify a specific age range or gender for participants. Therefore, participants of all ages and genders were included in this search. This review also only considered studies that focussed on participants who were amateur musicians or dancers. Studies that focussed on the wellness of professional, semi-professional, or retired musician/dancers, were excluded. Only studies that were written in English and published in the past 15 years were included. Systematic reviews and meta-analyses were excluded.

## Results

A total of 296 studies (excluding duplicates) were identified through the literature search process. Following the exclusion criteria, 268 were discarded resulting in 28 remaining studies (see Figure 1). Nine (32%) of the 28 studies examined the health and wellbeing effects of music and 19 (68%) studies focused on dance. Summaries of the music and dance studies included in the analysis are presented in Tables I and II. Within the music and dance categories, studies were grouped according to the focus of the research. The music studies examined wellbeing effects (including quality of life, social and mental health concepts, n = 7, 78%), or physiological cognitive health effects (n = 2, 22%) on participants. The dance studies were grouped similarly—those that evaluated wellbeing effects (including quality of life, social and mental health concepts, n = 7, 37%), and those that examined cognitive and physical (including physical activity) health effects (n = 12, 63%) of dance participation.

The results are presented according to the themes—wellbeing; cognitive health (music), or cognitive and physical health (dance)—for the music and dance studies separately. Within each theme, key concepts emerging through the review process are discussed.

### Music

The music research predominantly focused on wellbeing outcomes, used pre-test-post-test designs, and involved various musical activities and methodological approaches (see Table I). Of the nine studies collected, two observed chorale or singing participation, one was a rhythm-based intervention where participants engaged with group percussion experiences, one involved the use of instruments such as guitars, keyboard, and recorders, one involved group song-writing and music making experiences, two examined individual music lessons, and two were individual case studies of music making. Of these, four used pre-test-post-test study designs, one was a longitudinal study, and one was a randomized control trial. Results were acquired through various methods including surveys, cognition tests, interviews, and direct observations. The detailed summaries of the music research reviewed are in Table II.

#### Wellbeing

The research focusing on wellbeing outcomes through music participation focused on older adults and yielded mixed and often conflicting results. Cohen et al. (2006) conducted a multi-site, longitudinal study to observe the impact of a community-based chorale programme on participants’ morale, depression, social engagement, and general health. The programme was culturally appropriate, professionally conducted and targeted at older adults aged 65 and over in the USA. After one year of participation in a chorale programme, the intervention group (90 participants—77 at follow up) showed an increase in overall health and social activities, and decreases in prevalence of depression, reported falls and healthcare utilization compared to the non-chorale control group (76 participants—64 at follow up). Somewhat in contrast, Solé, Mercadal-Brotons, Gallego, and Riera (2010) found no substantial changes in quality of life (including physical and psychological wellbeing, and interpersonal relationships) in a sample of 83 people (average age ~72.6 years) participating in a variety of music programmes in Barcelona. Furthermore, although the authors did not observe an increase in overall health or social activities, they did suggest that this may have been because the participants already experienced high levels of quality of life and health at the outset. Solé et al. suggested that their results could be interpreted as evidence for maintenance, rather than improvement, of good quality of life through music participation. Yap, Kwan, Tan, Ibrahim, and Ang (2017) similarly found no statistically significant results in their randomized control trial (57 participants, 27 in control group) examining the impact of an 11-week long rhythm-focussed (group drumming) wellness programme on participants’ quality of life (measuring depressive symptoms, social isolation, and sleep quality). The lack of an increase or decrease in participants’ quality of life might indicate that the group drumming provided a means through which to maintain, rather than improve health.

Studies included also centred on the impact of group music participation on the social health of older people. Creech, Hallam, Varvarigou, McQueen, and Gaunt (2013) compared the experiences of people (500 participants) in a variety of community music groups (instrumental, song-writing, and chorale programmes) to those of participants in non-music groups, such as yoga, book clubs, and craft activities. They found that after nine months of participation, in comparison to baseline survey measures, those who participated in the musical activities reported higher levels of wellbeing than those who participated the non-music group activities. In a case study of older people in a community musical theatre group in Melbourne, Australia, Joseph and Southcott (2014) found that participation in the group provided its members with a sense of purpose and fulfilment, which they attributed to good quality of life and positive wellbeing.

One study examined the use of music to help regulate behaviour and promote active engagement in self-regulation in children. In Kim et al.’s (2006) pilot study, 35 adolescent Korean girls aged 11–12 years participated in a variety of music activities with the goal being to promote and improve their self-perception of control in relation to emotional regulation and behaviour. The results from this study were mixed. Only 5 of the 35 participants reported improved self-control and emotional regulation, however programme facilitators noted a much higher success rate than what was reported by the participants. The discrepancy in the research findings highlights the need to carefully consider the efficacy of objective and subjective self-report approaches when evaluating wellbeing outcomes of various interventions.

One study focused on music in a developing country to enhance population health and wellbeing. McConnell (2016) employed an interview-based ethnomusicological approach (126 participants) to investigate the use of music to facilitate communication about positive health behaviours in The Gambia. McConnell suggested that music activities helped to create social capital and build on new and existing social relationships in a way that was culturally appropriate in order to encourage healthy behaviour changes. An important example used in this paper was a song written and disseminated in 2014 detailing important health information about the Ebola epidemic in West Africa (including symptoms and how the disease is spread). Performance and teaching of this song encouraged trust and bonding between people which resulted in the overall message being much better received.

#### Cognitive health

Two studies met the inclusion/exclusion criteria and focused on cognitive health outcomes for older adults through short- and long-term music participation. Bugos, Perlstein, McCrae, Brophy, and Bedenbaugh (2007) recruited 39 older adults (60–85 years) to participate in one 30 minute individual piano lesson per week plus 3 hours of individual practice time. Their aim was to assess any changes in the participants’ performance on cognitive attention and working memory tests. The results were generally positive, finding that both memory and cognitive attention were improved and sustained over the 6 month intervention. Not only did the results show improvement in cognitive tasks related specifically to piano playing, but they also found that the skills gained in the intervention were transferrable to other, non-musical activities. Transferability across cognitive tasks is a major challenge when it comes to cognitive ageing interventions where participants are rarely able to transfer skills from one task to an unrelated activity. In a study of 70 older amateur musicians, Hanna-Pladdy and MacKay (2011) found that the longer and more actively an individual participated in musical activities throughout life, the better they performed on cognitive tests. Individuals with a long, active history of amateur musicianship (>10 years) performed better on executive processes, naming, and non-verbal memory tasks than their lower activity counterparts (1–9 years). Hanna-Pladdy and MacKay suggested that these results indicated a strong predictive effect of musical activity on safeguarding cognitive function in later life. Although, further research is needed to determine whether the result was due to the musical activity itself, or the type of individual with particular cognitive traits who was also drawn to musical activity in the first place.

#### Music summary of key findings

Overall, the research suggests that active music participation promotes the maintenance or improvement of sound wellbeing and health. The dominant research focus on older adults points to an apparent connection between social, group-based music participation and positive wellbeing outcomes for older people; supporting the notion that active engagement in socio-cultural performing arts practices represents an important social determinant of health. Further evidence suggests that music participation can contribute to building social capital as well as combating social isolation, which is important to good wellbeing and health across cultures and age-groups. The impact that cognitive health can have on wellbeing and quality of life cannot be ignored. A person’s functional capacity and their ability to positively engage in the world around them can heavily influence their wellbeing. Especially in old age, an individual’s capacity to maintain healthy social practices may be inhibited by poor cognitive health and function. The ability to improve cognitive health through active music participation is important in maintaining positive wellbeing in individuals and populations. As the research predominantly involves older adults and Western cultures, further studies are needed to fully understand the contribution of music participation to wellbeing and health for “well-functioning” individuals and populations across cultures and age groups.

### Dance

The dance studies reviewed were more unified in their approach and their results than the music studies (see Table III). Of the 19 studies identified, six focused on the effects of partnered dance (including ballroom dancing and tango) whilst 13 involved group dancing of some form. Most studies involved a type of experimental design and predominantly quantitative data generation techniques, including physical tests, surveys, and interviews. Two studies involved participant observation. The detailed summaries of the dance research reviewed are in Table IV.

#### Wellbeing

The contribution of dance participation to wellbeing involves a variety of social, physical, and personal components. However, most prominent is the overarching quality of life, or sense of life satisfaction group-based dance participation brings. Through a randomized control trial involving 57 women aged 65–80 years in Portugal, Cruz-Ferreira, Marmeleira, Formigo, Gomes, and Fernandes (2015) observed an increase in life satisfaction and physical fitness in the intervention group when compared to their control group after 24 weeks. They concluded that the integration of mobility, physical, cognitive, and social skills attributed to the dance classes was the root cause for this improvement in quality of life. Muro and Artero (2017) studied 87 young women (mostly university students; average age 20.88 years) who participated in at least 3 hours of non-competitive dance practice every week. The aim of this cross-sectional study was to determine if wellbeing and life satisfaction could be correlated with dance participation. Muro and Artero found that those who regularly participated in dance practice were more mindful and experienced a better quality of life than those who did not dance (or practice any other kind of sport). Nadasen (2008) too found that a group of 30 older women participating in regular dance classes reported a significant increase in their engagement in social activities and a widening of their social networks. For most of these women (aged 60–82 years), having joined the dance class merely as a way of boosting their weekly physical activity, the boost to their social activities (and their quality of life) was unexpected but welcome.

Partnered tango dancing has been found to facilitate a state of mindfulness, which has important implications for mental health, in addition to promoting psycho-social-emotional and physical benefits. In a study of 110 non-competitive dancers of tango Argentino in the Netherlands and Germany (average age 37.96 years), Kreutz (2008) found that participants were not only motivated by the physical benefits of the activity, but also by the emotional, psychological, and social benefits. He observed that, compared to participants in other activities (dance-related and not), the tango dancers were often more practiced in mindfulness and awareness both of themselves and others around them. Pinniger, Brown, Thorsteinsson, and McKinley (2012) conducted a pre-test-post-test randomized control trial to examine the impact of dance participation on stress reduction. Sixty-six participants were assigned to an Argentine tango dance class, a meditation class, or a waitlist control group. The results revealed that the tango group showed significant reductions in their stress levels and increases in reported levels of mindfulness when compared to the meditation and waitlist control groups.

Dance participation can also contribute to positive body image and self-acceptance. Moe (2014) analysed a series of 67 semi-structured interviews with women (>50 years) participating in belly dancing in the US. She found that many participants’ experiences of their own body-image (particularly as they aged) were improved by their participation in belly-dancing. She found that the building of community and participating in such a sensual art form had a positive impact on the participants’ relationships with their bodies and they reported feeling more emotionally and psychologically comfortable with themselves. Duberg, Moller, and Sunvisson (2016) found similar results in a randomized control trial of 112 teenage (13–18 years) girls. The girls in the intervention group reported dance classes as a safe, enjoyable space where they could express themselves emotionally. Improved self-image and self-trust were paramount to the perceived quality of life and wellbeing of the participants.

#### Cognitive and physical health

The research reviewed suggests that dance participation makes an important contribution to the cognitive and physical health of older adults. Coubard, Duretz, Lefebvre, Lapalus, and Ferrufino (2011) conducted a study comparing the cognitive and physical benefits to 65–83 year old adults of participation in contemporary dance to tai chi and fall prevention classes (110 participants). Following a 5.7 month period of participation, dance was the only activity that improved attention switching and cognitive flexibility in older participants. Similarly, Jan-Christoph, Izabela, Tobias, and Hubert (2010) found, via a pre-test-post-test study, that the amateur dance intervention group (with 62 participants aged 61–94 years) showed better performance on cognitive tests as well as motor performance, posture and balance exercises, and reaction times compared to the non-dance control group. In an intervention study of 35 older people, Kattenstroth, Kalisch, Holt, Tegenthoff, and Dinse (2013) also found that participants in the dance class intervention group showed improvements in cognition, attention, motor skills, posture, reaction time and self-reported wellbeing.

The results of research focussing on dance participation as a means for older adults to maintain posture and balance was positive, yet the impact of dance on fall prevention was mixed. Rahal et al. (2015) conducted a randomized control trial of 76 participants aimed at assessing the impact of two group-based movement interventions (one a ballroom dancing group, the other tai chi) on older adults’ (>60 years) balance post intervention. They found that both ballroom dancing and tai chi improved balance performance, but on different tests. For example, the ballroom dance group demonstrated better balance standing on one leg with eyes closed than the tai chi group, but the tai chi group was better at static balance standing on two legs. Filar-Mierzwa, Dlugosz, Marchewka, Dabrowski, and Poznanska (2017) also found that a dance class programme for 24 women (>60 years) significantly improved their balance. While good balance is important for preventing falls, the efficacy of dance used as a tool aimed specifically at preventing falls in older people seems to be contested. Granacher et al. (2012) found that salsa dancing improved static postural control and gait patterns in twenty-four 63–82 year-old participants, potentially reducing falls. However, Merom et al. (2016) reported that participation in dance programmes did nothing to reduce falls in its participants of a similar age (530 participants across 23 retirement villages). The capacity of older individuals to balance was linked to the strength of their bodies and their ability to move unaided.

The positive impact dance participation can have on individuals’ physical activity and health appear to be optimal when the style and context of the activity are culturally and socially appropriate. Pacheco, Hoyos, Watt, Lema, and Arango (2016) and Dewhurst, Nelson, Dougall, and Bampouras (2014) both investigated the efficacy of different genres of dance to improve physical fitness in women aged 60 years and over. Both found dance to be an appropriate and effective method for improving physical health in older women when the dance style was matched to the cultural and social environment. Dewhurst et al. focussed on Scottish country dancing with 60 women in Scotland whilst Pacheco et al. examined Columbian Caribbean folk dancing with 27 women on the Caribbean coast of Columbia. Murrock and Gary (2008) too investigated the use of culturally specific dance programmes with 126 African American women to improve physical fitness and functional capacity. The researchers observed significant positive effects after 18 weeks of dance classes in the intervention group as compared to the non-dancing control group. Similarly, Vahabi and Damba (2015) found that a gender-specific, Bollywood-styled dance programme was effective in promoting physical health for 27 South Asia immigrant women (average age 42 years) living in the Greater Toronto Area, in Canada.

One study involving children found that dance participation promoted physical health. Huang, Hogg, Zandieh, and Bostwick (2012) implemented a ballroom dancing programme at two elementary schools in New York City (79 participants) as part of an arts-in-education programme (separate from the usual physical education programme). Ballroom dancing participation increased the time involved in physical activity, heart rate, and provided an alternative and creative opportunity for children to be active during their school day.

### Dance summary of key findings

Dance participation appears to contribute positively to individuals’ wellbeing and health across cultures and age groups. It seems to provide a safe context for social engagement and building communities, which crucially enables participants to construct and maintain their own wellbeing and health in a range of ways: cognitive function, physical health, stress reduction, self-perception and mental health. The research has focussed on older adult women and the contribution of social, group-based dance participation to sound cognitive and physical health, and experiences of wellbeing and quality of life. However, a good proportion of the studies involved younger and middle-aged adults, again mostly women. Males appear to be underrepresented in the literature. Only partnered dance interventions seem to involve males (adults or children). This indicates an opportunity to expand the research to examine the particular wellbeing and health affordances of dance participation beyond female populations. While the majority of the literature seems to indicate that dance participation can contribute to healthy ageing, far more is needed to confirm or refute assumptions. For example, falling is a serious health risk that is regularly linked to old age, making a significant proportion of reasons older people access emergency healthcare facilities in Australia (Merom et al., 2016). Fall prevention is therefore a high priority when it comes to public health prevention (Fernandez-Arguelles, Rodriguez-Mansilla, Antunez, Garrido-Ardila, & Munoz, 2015). Mobility and good physical health make important contributions to an individual’s sense of autonomy and confidence to maintain overall health. For example, having the ability to cook and clean unassisted, or maintain social health by being able to attend social events and functions without difficulty. Further research is also needed to more fully understand the impact of dance participation in younger populations, including children, and how this might facilitate individuals to self-manage their wellbeing and health across the life-course. The evidence suggests that dance participation affects individuals in a range of ways that can improve health determinants, such as stress and social capital, which can lead to an improvement in overall wellbeing and health.

## Discussion

The results of this systematic review indicate that participation in music and dance performing arts is effective for maintaining and promoting wellbeing and good health within a population. In addressing the initial key research question, the evidence suggests that music and dance relate to several social determinants of health in potentially positive ways. For example, music and dance participation can improve social determinants of health, such as stress, social isolation, autonomy, and social capital, and maintain or enhance individual wellbeing. Socio-cultural performing arts practices that enable active participation, such as music and dance, provide people with positive and creative ways of engaging with their communities and challenging personal physical, cognitive, and emotional systems. Engagement in music and dance potentially offers participants a range of wellbeing and health benefits that are unsurpassed by other activities, such as tai chi, writing, and sport. Music and dance activities appear to be highly engaging and enjoyable, indicating that participants might be positively motivated to commit to ongoing participation in music and dance interventions or programmes. Furthermore, music and dance activities can be delivered and accessed with relatively little expense in comparison to mainstream healthcare facilities and services. The evidence reviewed here suggests that music and dance activities enhance participants’ physical, cognitive, and social health and wellbeing across all groups, and interventions can be highly effective and able to be adequately maintained.

The second research question sought to identify gaps in the current body of research examining how healthy individuals/populations maintain and promote good health and wellbeing through music and dance participation. The primary gap related to participant demographics. The majority of the studies reviewed involved older participants, in both the music and dance categories. Many studies only involved people over the age of 60 years, potentially reflecting a strategic research agenda of governments around the world facing an ageing population that presents an increasing drain on the economy (AIHW, 2018). Music and dance are increasingly being associated with creative ageing practices and explored as cost-effective means for older adults to improve social, cognitive, and physical health (Hanna, 2013). Although this is a positive research focus, it comes at the expense of a depth of research with children and young-middle-aged adults, who are missing out on the potential benefits of these interventions. Childhood and adulthood are critical periods of life in which to establish participation in activities that are going to assist people to maintain their wellbeing and health into old age. Future research should prioritize developing the body of research across all age groups regarding the wellbeing and health affordances of participation in cultural activities in order to assist individuals/populations to recognize opportunities and develop skills to enhance and maintain their sense of wellbeing now and moving into the future.

There exists a gender disparity in the research reviewed with female participants outnumbering male. Whilst men were generally not excluded, they were not well represented in the music or dance studies. It is important that both men and women’s experiences are identified and discussed equally. Women might feel more comfortable participating in cultural activities, particularly in Western-centred cultures. However, the physical, cognitive, emotional and social benefits from active participation in music or dance are arguably similar for all genders. Future research should attempt to build engagement with current minorities to understand how music and dance participation enhances the wellbeing and health of entire populations.

A large and unexpected gap in the research was the lack of representation of minority and Indigenous groups. There were no studies that looked at the benefits of music and dance to Indigenous peoples or communities. Nor were there any studies that focussed specifically on LGBTQIAP+ communities and their engagement in music and dance activities. Only one study (Vahabi & Damba, 2015) examined the benefits of dance participation for a migrant population. This lack of representation of minority and Indigenous groups might, however, be explained by the exclusion criteria, which removed studies that involved participants who had pre-existing medical conditions. Indigenous communities, particularly are known for having much poorer health than their none Indigenous counterparts (Gracey & King, 2009). LGBTQIAP+ and migrant populations too are known for their poorer health outcomes (Lick, Durso, & Johnson, 2013; Viruell-Fuentes, Miranda, & Abdulrahim, 2012). Increasingly, research is supporting the notion that social determinants of health include concepts such education (Shankar et al., 2013), sexual orientation (Logie, 2012) and cultural practices (King, Smith, & Gracey, 2009). It is recommended that further research is needed to investigate how minority and Indigenous groups’ wellbeing and health might benefit through active participation in culturally appropriate music and dance activities.

A crucial gap in the literature is the lack of theory with which to organize and explain the current research results and formulate predications for future research. Without such theory, little can be concluded as to the exact mechanisms that might make culturally situated music and dance participation such effective tools for personal management of sound wellbeing and health, or purposeful health intervention strategies. Taking a systems theory approach considering the complexity of interrelatedness of components (Kannampallil, Schauer, Cohen, & Patel, 2011) might provide a useful framework through which to consider the intrapersonal psychological, emotional and physical, and interpersonal facets afforded by music and dance participation, embedded within a particular socio-cultural context. Developing testable theoretical frames is crucial for future research to clearly understand how and why music and dance practices are effective. This is also of critical importance for generating a strong evidence base linking performing arts cultural practices to wellbeing and health with significant implications for contemporary healthcare practice and policy.

In addressing the third research question—the current body of literature lacks clear theoretical bases and consistent methodological approaches, which creates challenges for clear recommendations for healthcare and policy. However, this also signifies research opportunities moving into the future. The general lack of consistency between study designs (particularly in relation to the music studies), and some conflicting evidence points to the lack of coherent theory and allied methodology employed in studies examining music and dance participation for wellbeing and health outcomes. As is usually the case in a new field, the research tends to be exploratory, and employs more ethnological, phenomenological, narrative-based approaches, which holds little authority when it comes to influencing healthcare policy and practice. To be better understood and utilized by healthcare professionals and policymakers, stronger theoretical and methodological research bases must be developed to enable more systematic analysis across studies.

The lack of unity in research approaches and methodologies perhaps reflects the challenges inherent in melding two disciplines—art and science—underpinned by contrasting epistemological and ontological stances. The literature reviewed indicates a difficulty in matching the scientific research approach with the relatively unstructured, or constructivist nature of music and dance. Health sciences, and by extension health policy and research, require evidence that interventions are replicable, scalable, cost effective, and maintainable or else they are ineffective and disregarded. Art programs, on the other hand, tend to be more flexible and responsive to the participants and the context at any given time. The outcomes for participants in an art programme at a given time and place cannot be guaranteed and, therefore, might not be replicable, which highlights the contention between the two disciplines. Moreover, the research sample size is a key consideration as what might be acceptable for a health intervention and what is acceptable for an arts intervention can differ significantly. For example, a number of the reviewed studies cite small sample sizes as a limitation to their validity and suggest more research needs to be done with larger samples. In terms of a health science intervention, larger sample sizes are favoured to confidently establish validity, replicability and generalizability of the intervention. However, most community music and dance groups and programmes will often have no more than 30 people involved, depending on the type of group (for example a community orchestra may have up to 120 members whereas a community dance troupe might have only 12 members). The size of the group can impact the range and type of activities possible. From a music or dance perspective, a small sample size might be essential for the efficacy of an intervention or programme. Music and dance health interventions need to recognize this difference and account for it in how they communicate their research.

The research reviewed here seems to reflect the challenge in attempting to apply a health science lens and platform to examine and communicate the wellbeing and health affordances of music and dance participation, whilst maintaining the fluid and humanistic qualities that characterize the art forms. A new interdisciplinary middle ground must be developed, with theory and research approaches, and a language for effective communication that honours the traditions of each discipline whilst creating a new mould. The emerging arts health discipline is forging new ground in this respect. Effective collaboration and communication between those working across arts and health disciplines and healthcare is paramount in continuing to move forward and make meaningful changes to the cultural health of individuals and populations.

The results of this review need to be considered in light of the purpose and inclusion/exclusion criteria. We identified a small body of research examining the wellbeing and health outcomes for participants of music and dance performing arts practices. However, a much larger body of research is developing that is focused on using arts practices, such as music and dance, in the treatment of ill-health. In addition, this review was limited to research into music and dance participation, excluding other art forms such as writing, drama, and visual art, or attendance at cultural events as an observer. As the inclusion and exclusion criteria negated studies with “unhealthy” participants, this potentially biased the results against certain groups, such Indigenous populations. Because of the complex nature of Indigenous health, many Indigenous participants would have been excluded from reviewed literature. This review is limited to the approaches and concepts identified in the research examined, and according to the definitions of music and dance performing arts applied. For Indigenous populations and those from non-Western cultures, this review might not have identified how music and dance concepts relate to other concepts that are crucial for wellbeing and health. Further research is needed to fully understand how all art forms might impact the wellbeing and health of all individuals/populations to best inform public health policy and practices in the future.

## Conclusion

The results of this review suggest that actively participating in music and dance is an effective means through which individuals and populations can maintain and promote wellbeing and health across the life course. Performing arts participation crucially relates to social determinants of health, particularly from the perspective of building social and cultural capital, encouraging healthy behaviours such as physical exercise and management of stress and mental health, and reducing social isolation. Furthermore, performing arts programmes can and are being delivered using existing infrastructure making them potentially cost-effective ways of assisting people to self-manage their good health and wellbeing and reducing burden on traditional healthcare systems. Shifting understandings of what good health actually means to both individuals and populations suggests that health professionals and policymakers might too need to review the concept of what it means to be healthy and how to best support people to manage their wellbeing and health.

There are a number of key opportunities to build and shape the body of research for a future, broader impact on the health discipline. Major gaps in the research relating to age, gender, and minority and Indigenous groups need to be considered and more research is needed in order to ensure the best outcome for all groups. More research is needed to target people in their middle years of life and children, as well as the elderly. Targeting these groups will ensure that the protective health effects of music and dance can carry on into old age, and future policy needs to reflect this.

It is recommended that research at the nexus of arts and health begin to develop theoretical and methodological frames, and a language for effectively communicating with arts and health disciplines. This is crucial to enable important findings to be translated into policymaking and healthcare practice. While seemingly dichotomous disciplines, this review has highlighted that there are considerable connections and opportunities to bridge the disciplines and potentially enhance the health and lives of many.

## Geolocation information

Mary Broughton: https://researchers.uq.edu.au/researcher/810
